# Assessing Readiness for Change: A Baseline Situational Analysis of Breastfeeding Support Within Acute and Community Healthcare Settings in the Republic of Ireland

**DOI:** 10.1111/mcn.13778

**Published:** 2025-04-16

**Authors:** Helen Mulcahy, E. Lehane, Michelle O'Driscoll, Kathleen McLoughlin, Catherine Buckley, Elizabeth McCarthy, Sandra O'Connor, Mairead O'Sullivan, Elizabeth Heffernan, Margaret Murphy, Rhona O'Connell, Patricia Leahy‐Warren

**Affiliations:** ^1^ School of Nursing and Midwifery University College Cork Cork Ireland; ^2^ School of Medicine University of Limerick Limerick Ireland; ^3^ Northridge House Education & Research Centre St. Luke's Home Cork Ireland; ^4^ School of Nursing & Midwifery Trinity College Dublin Dublin Ireland; ^5^ Centre of Nursing and Midwifery Education University Hospital Kerry Tralee UK

**Keywords:** breastfeeding, clinical practice, health services research, healthcare facilities, healthcare policies, maternal and child health services, organisational culture

## Abstract

Breastfeeding is a critical component of maternal and child health, but breastfeeding rates vary widely, with Ireland's rates lowest in Europe. This paper, the first stage of the Practice Enhancement for Exclusive Breastfeeding (PEEB) study, describes the methodology and key findings from a multi‐component baseline situational analysis of breastfeeding support conducted in acute and community healthcare settings in Ireland. Guided by the Promoting Action on Research Implementation in Health Services (PARIHS) framework, a survey of healthcare professionals (*n* = 85) examined breastfeeding training, competency and attitude towards practice change. Additionally, a workplace environment and policy assessment were conducted. A gap between current practices and evidence‐based guidelines was demonstrated and the need for cultural shifts within healthcare settings towards better breastfeeding support. The study also uncovered notable variations in breastfeeding training, perceived barriers to effective breastfeeding support, including staff shortages and communication challenges, which impede successful implementation of breastfeeding initiatives. Despite high levels of staff motivation, there was a lack of empowerment and confidence to implement change. Findings revealed significant differences between community and acute staff across certain factors like community staff being less likely to agree that their teammates considered implementation of changes as futile, acute staff were less likely to disagree that changes would be subject to audit. The PARIHS framework provided a structured approach to understanding the evidence and contextual factors relevant to implementing evidence‐based practices in breastfeeding support. Future work will focus on the design and implementation of strategies to optimise breastfeeding support across acute and community settings.

## Summary

- Understanding and addressing the unique challenges of acute and community settings is crucial to optimise breastfeeding support and outcomes. The PARIHS model offers a useful theoretical framework to identify evidence and contextual factors relevant to enhancing support and tailored interventions.
- Differences in training, attitudes and perceived barriers between acute and community healthcare staff require setting‐specific strategies to enhance breastfeeding support.
- A cultural shift within healthcare, supported by comprehensive training and education, is vital to align practice with evidence‐based guidelines.
- Addressing systemic barriers, such as staffing and empowering healthcare staff through confidence‐building measures are essential steps towards effective change implementation.

## Graphical abstract

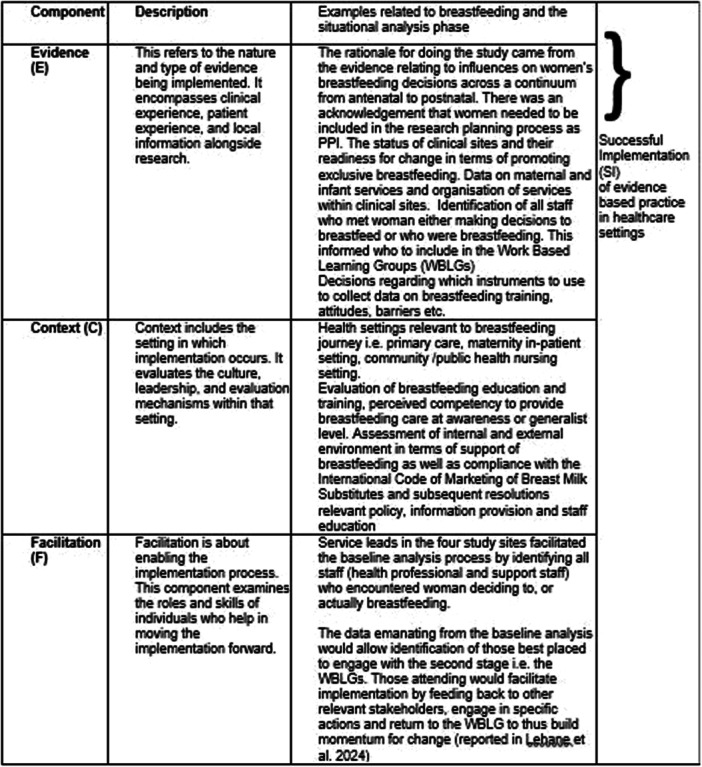

## 1 Introduction

Exclusive breastfeeding is accepted as a significant determinant of infant and maternal health, associated with reduced infant mortality and morbidity, alongside numerous long‐term health and economic benefits (Baker et al. 2023; Pérez‐Escamilla et al. 2023). Despite global health policy advising exclusive breastfeeding for the first 6 months postpartum and up to 2 years and beyond with complementary foods (World Health Organization 2001), compliance rates vary across nations (Rollins et al. 2023). Few countries are likely to meet their target of exclusive breastfeeding to 70% by 2030 with global rates currently standing at 49% (Pérez‐Escamilla et al. 2023). The World Health Assembly's initial target of exclusive breastfeeding of 50% by 2025 might be reached, but estimations show that the goal of 70% by 2030 might not be attainable. At the current rate, only 61% of children will be exclusively breastfed by 2030. In Ireland, breastfeeding rate at birth was only 64% in 2023, which means only 64% of babies receive breastmilk at their first feed following birth (HSE Breastfeeding Action Plan Implementation progress report), and only 36.9% are exclusively breastfed on discharge at Day 3 of life (National Women and Infants Health Programme 2021).

In 2023, the prevalence of any breastfeeding (exclusive and nonexclusive) in Ireland reduced from 63.1% to 60.3% (Department of Health 2023) when the mother–infant received their first visit from a public health nurse which is around 72 h of discharge from the maternity unit. The prevalence further drops at 3 months postpartum, which is about 42% of mother–infant dyad (this reflects exclusive and nonexclusive breastfeeding), with most of those (32%) exclusively breastfeeding. It is not possible to calculate the prevalence of exclusive breastfeeding below 6 months in Ireland as routine data are not collected beyond 3 months. Thus, there is an imperative need for tailored interventions to enhance the practice of healthcare professionals to promote exclusive breastfeeding throughout the perinatal journey. That journey begins at the initial confirmation of pregnancy with a General Practitioner (GP) and continues through antenatal, intranatal and postnatal care, extending into a seamless transition to community care.

Any duration of breastfeeding can be influenced by peer supporters, healthcare professionals and healthcare workers across the perinatal period in both hospital and community settings. There are several barriers to breastfeeding including lack of evidence‐based knowledge and skills and consistent follow‐up or predictability of care from healthcare professionals. Furthermore, awareness of, and support for, breastfeeding from all healthcare workers who come in contact with mothers is recommended (HSE, Lehane et al. 2024). Maternal decisions about infant feeding are made in the context of systems and policies. These include the maternity care system, professional training and public awareness (Rollins et al. 2023). An integral part of breastfeeding support programmes is education and training for all staff who provide breastfeeding support. This may include structured breastfeeding support programmes with multiple facets, such as the UNICEF/WHO Baby Friendly Hospital initiative (BFHI, Gavine et al. 2017). However, the concept of training in the context of breastfeeding support should be broadened, that is, extended from the healthcare professional domain to the public domain.

Irish policy (Department of Health and Children 2005; Department of Health 2016, 2019, 2021; HSE 2016) specifies all maternity service staff must be able to provide relevant evidence‐based breastfeeding support and for this support to continue into primary care. The maternity healthcare setting touch points for women encompass care led by a maternity hospital consultants or a shared care model delivered by general practitioner and consultant across antenatal to postnatal. While there are midwifery‐led and early postnatal discharge schemes in some locations, the shared care model is more common. Public Health Nurses provide most postnatal care on discharge from maternity units.

The effective translation of evidence into practice settings requires strategies to identify and optimise the existing infrastructure to include the environment, policy, staff knowledge and attitudes. Implementation science theory, which is the scientific study of methods to promote the systematic uptake of research findings and other evidence‐based practices (EBPs) into routine practice, is fundamental to this process to improve patient outcomes (Graham, Tetroe, and Pearson 2014; Harrison and Graham 2012; Lockwood et al. 2016; Parmelli et al. 2011).

**Figure 1:**
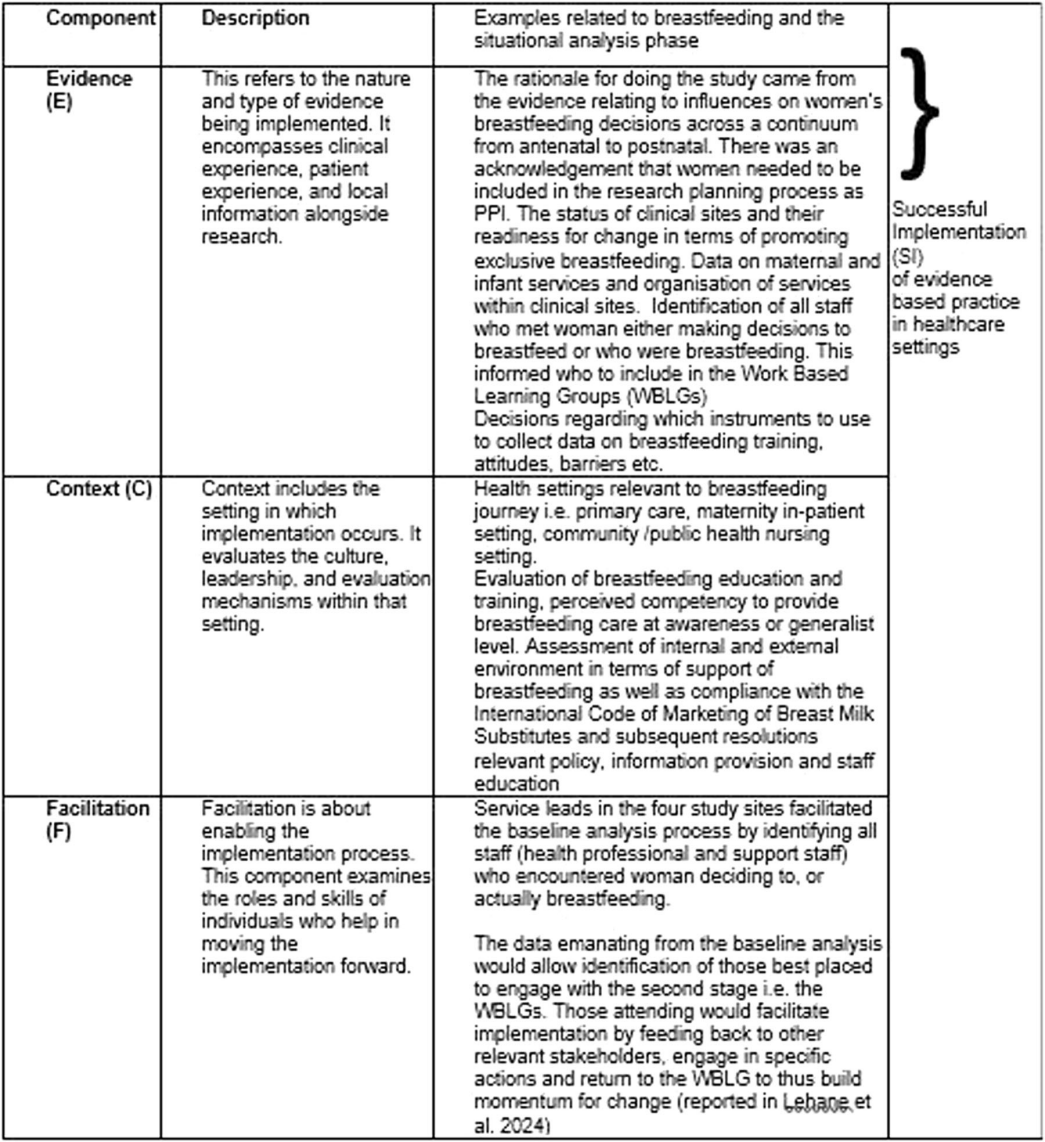
Overview of the PARIHS Framework (adapted from Kitson, Harvey, and McCormack 1998; Rycroft‐Malone et al. 2004; Rycroft‐Malone et al. 2002; Rycroft‐Malone 2004).

Implementation science frameworks, theories and models can help to explain how and why EBPs succeed or fail. One such model, the Promoting Action on Research Implementation in Health Services (PARIHS) framework, is designed to facilitate the application of evidence‐based knowledge into a range of healthcare settings (Kitson et al. 2008; Kitson and Harvey 2016; Laker et al. 2014). This model has been used widely to guide and analyse the implementation of knowledge into practice across settings, including women's health where it has been successfully used in family planning (Niemeyer Hultstrand et al. 2020), pregnancy care (Meloncelli, Barnett, and de Jersey 2020) and primary care health promotion (Strid, Wallin, and Nilsagård 2022). As outlined in Figure 1, PARIHS conceptualises successful implementation (SI) as a function of the nature and type of evidence (E), the quality of the context (C) and the effectiveness of facilitation (F) (Duan et al. 2022).

In this model, ‘evidence’ includes all knowledge sources including research studies, clinical guidelines, expert opinions and patient preferences. ‘Context’ is the environment where evidence is applied and encompasses leadership, culture, evaluation methods and openness to innovation. Finally, ‘facilitation’ is both a role and process involving guidance, support and help provided to clinical teams to adopt new practices and knowledge. In addition, Figure 1 specifically relates ‘evidence’, ‘context’ and ‘facilitation’ to breastfeeding and their application to the overarching Practice Enhancement for Exclusive Breastfeeding (PEEB) study.

The PEEB study was designed to enhance breastfeeding support provided to women, partners and infants throughout the perinatal journey. The first step of this multiphase study involved a situational analysis. This involved a baseline assessment of environmental readiness, existing policy, staff competence, attitude and their readiness for change. This paper presents the methods and key findings from the situational analysis conducted in primary and secondary healthcare settings relevant to the perinatal journey of women. To this end, we sought to augment the development of effective and sustainable exclusive breastfeeding strategies.

## 2 Methods

### 2.1 Design

Guided by the PARIHS framework (Kitson, Harvey, and McCormack 1998; Rycroft‐Malone et al. 2004; Rycroft‐Malone et al. 2002; Rycroft‐Malone 2004), the PEEB study employed a Participatory Action Research (PAR) design (Baum, MacDougall, and Smith 2006) and was conducted in four distinct clinical settings within a region of Ireland. A baseline situational analysis assessed staff demographics, education, skill‐based training, breastfeeding competency and attitude towards practice change, as well as a review of the environment and policy, using both quantitative and qualitative data collection methods. These findings informed the direction of the Work Based Learning Groups and subsequent development of breastfeeding support strategies (Lehane et al. 2024).

### 2.2 Context

The study was conducted across both acute and community healthcare settings, including a general hospital providing maternity services, two General Practitioner (GP) practices and one Primary Care Facility, which includes public health nurses and allied health professionals. The maternity study sites were selected based on existing clinical‐academic partnerships within a Regional Health Learning System. This maternity unit is the only one providing a service in this geographical county. Therefore, all maternity admission discharges would be confined to one county which was advantageous. The public health nursing service and the two GP sites were conveniently located in or near the town in which the maternity unit is located. These site locations, although limited by convenience, were necessary as staff from them would be required to participate in Work Based Learning Groups over a period of a year as discussed in another publication (Lehane et al. 2024). Breastfeeding support infrastructure at the four chosen sites is typical of maternity healthcare services across Ireland. Two researchers conducted the environmental assessment of the four sites. Participation from staff was sought through a recruitment poster in each of the study sites. Any staff member that a woman encountered who could influence her breastfeeding experience was deemed eligible to participate. A convenience sample of healthcare staff across each study site was recruited via information posters to complete study paper questionnaires which were returned anonymously into locked boxes.

### 2.3 Ethical Issues

Ethical approval was granted by the Clinical Research Ethics Committee of the Cork Teaching Hospitals (ECM 4 (ee) 10/8/2021 and ECM 3 (c) 19/10/2021). There were no significant areas of ethical concern in this study.

### 2.4 Data Collection Instruments

The instruments used to collect data for the baseline situational analysis are as follows:

1. A *Physical Environmental Assessment Checklist* (World Health Organization 2009) was used by two researchers to assess the internal and external environment of each of the four participating sites. External assessments related to the setting and parking, while internal assessments examined specific designated feeding areas, aesthetics, seating and breastfeeding signage. In addition to the physical environment, the tool considers compliance with the International Code of Marketing of Breast Milk Substitutes (the Code) and subsequent resolutions (World Health Organization 1981), relevant policy, information provision and staff education.
2. A *staff survey* composed of 70–97 items (dependent on response pathway). Nine items were questions designed to capture demographic characteristics, training and staff engagement with both breastfeeding mothers and babies (Table 1). The survey also included two validated instruments: (a) *VOCALISE*, a 20‐item questionnaire to measure staff perception of barriers to change (Laker et al. 2014) and (b) the 23‐item *EBIQ* to assess staff perceptions of clinical and patient experience, research utilisation and context (Bahtsevani and Idvall 2016; Bahtsevani et al. 2008). Finally, a tool, adapted from Gallagher et al.'s (2015) breastfeeding competency framework, was included in the survey to allow measurement of perceived competency (and the associated sub‐domains of staff knowledge, skills, attitude, and behaviour). Within the competency section, staff selected generalist level if they deemed themselves as ‘actively engaged in providing regular care and support to breastfeeding mothers’. Further questions asked if they were engaged full‐time in this role and if they provided in‐depth care and support with breastfeeding challenges. Those not engaged full‐time in the care of breastfeeding mothers but who had some orientation in supporting breastfeeding mothers and children completed the ‘awareness level’ items. Respondents rated their perceived competency across each indicator statement using a five‐point Likert scale (1 = Strongly Agree and 5 = Strongly Disagree).

**Table 1:** Staff demographics, role, level of engagement and training in breastfeeding.

	Hospital *n* = 51 (60%)	GP site 1 *n* = 8 (9.4%)	GP site 2 *n* = 11 (12.9%)	Primary Care Centre *n* = 15 (17.6%)	All *n* = 85 (100%)
Age (years)					
< 30	16 (31.4)	0 (0)	0 (0)	1 (6.7)	17 (20)
30–39	9 (17.6)	4 (50)	3 (27.2)	5 (33.3)	21 (24.7)
40–49	17 (33.3)	2 (25)	4 (36.4)	5 (33.3)	28 (32.9)
50–59	8 (15.7)	2 (25)	2 (18.2)	4 (26.7)	16 (18.9)
60+	1 (2.0)	0 (0)	2 (18.2)	0 (0)	3 (3.5)
Gender					
Female	41 (80.4)	8 (100)	8 (72.7)	15 (100)	72 (84.7)
Male	9 (17.6)	0 (0)	1 (9.1)	0	10 (11.7)
Not reported	1 (2)	0 (0)	2 (18.2)	0	3 (3.6)
Education level					
Secondary school	4 (7.8)	1 (12.5)	1 (9.1)	0 (0)	6 (7.1)
Degree	19 (37.3)	1 (12.5)	2 (18.2)	3 (20)	25 (29.4)
Masters	16 (31.3)	1 (12.5)	0 (0)	3 (20)	20 (23.5)
Postgraduate qualification	7 (13.7)	4 (50)	3 (27.2)	9 (60)	23 (27.1)
Professional qualification	5 (9.8)	1 (12.5)	5 (45.4)	0 (0)	11 (12.9)
Role					
Midwife	16 (31.4)	0 (0)	0 (0)	0 (0)	16 (18.8)
Nurse—Hospital	13 (25.5)	0 (0)	0 (0)	0 (0)	13 (15.2)
Nurse—Community	0 (0)	2 (25)	0 (0)	3 (20)	5 (5.9)
Doctor Hospital Community	3 (5.9)	0 (0)	0 (0)	0 (0)	3 (3.5)
Doctor—Community	0 (0)	3 (37.5)	3 (27.3)	0 (0)	6 (7.2)
Public Health Nurse	0 (0)	0 (0)	0 (0)	5 (33.3)	5 (5.9)
Student Doctor	6 (11.8)	0 (0)	1 (9.1)	0 (0)	7 (8.2)
Student Nurse	2 (3.9)	0 (0)	0 (0)	0 (0)	2 (2.3)
Student Public Health Nurse	0 (0)	0 (0)	0 (0)	3 (20)	3 (3.5)
Other allied healthcare staff	9 (17.6)	0 (0)	0 (0)	4 (26.6)	13 (15.3)
Non‐healthcare staff	2 (3.9)	3 (37.5)	7 (63.6)	0 (0)	12 (14.2)
Time in current role (years)					
Up to 1	12 (23.5)	2 (25)	2 (18.1)	3 (20)	19 (22.3)
2–5	11 (21.6)	2 (25)	3 (27.2)	2 (13.3)	18 (21.2)
6–10	8 (15.6)	1 (12.5)	1 (9.1)	2 (13.3)	12 (14.1)
11–20	15 (29.5)	2 (25)	4 (36.4)	6 (40)	27 (31.7)
> 20	5 (9.8)	0 (0)	1 (9.1)	2 (13.4)	8 (9.5)
Not specified	0 (0)	1 (12.5)	0 (0)	0 (0)	1 (1.2)
Level of engagement					
Actively engaged	20 (39.2)	1 (12.5)	2 (18.1)	8 (53.3)	32 (37.6)
Not engaged full‐time	24 (47.1)	6 (75)	9 (81.9)	6 (40)	45 (53)
In‐depth care	4 (7.8)	0 (0)	0 (0)	1 (6.7)	5 (5.8)
Yet to have opportunity to engage	1 (1.9)	0 (0)	0 (0)	0 (0)	1 (1.2)
Not specified	2 (3.9)	1 (12.5)	0 (0)	0 (0)	2 (2.4)
Training in last 5 years					
Yes	25 (49.0)	0 (0)	1 (9.1)	8 (53.3)	34 (40.0)
No	23 (45.1)	5 (62.5)	9 (81.8)	6 (40.0)	43 (50.6)
Informal experience	3 (5.9)	3 (37.5)	1 (9.1)	1 (6.7)	8 (9.4)

### 2.5 Units of Study

The units of study encompassed four clinical sites with a total of 85 participants. These participants were categorised based on their profession, education level, age and level of engagement with breastfeeding practices (Table 1). For the purposes of statistical analysis to compare differences between settings, the General Hospital is referred to as ‘acute setting’ and the two GP sites and Primary Care Facility are referred to collectively as ‘community healthcare settings’.

### 2.6 Data Processing

Data were analysed using SPSS v29.0.1.0 (171) to produce descriptive and inferential statistics as appropriate to the data set. On the VOCALISE tool, it is important to note that items 1, 4, 6–14, 17 and 18 are reverse scored and data were transformed at the point of processing.

## 3 Results

### 3.1 Physical Environmental Assessment

In terms of the environmental assessment with regard to accessibility, one community site had restricted buggy access and lacked appropriate parking, while the other three sites were fully accessible with suitable parking. There was no welcoming signage for breastfeeding in waiting areas, except for the hospital, which offered facilities for hand‐pumping, expressing and storing breastmilk. None of the four sites had designated breastfeeding rooms, and one of them lacked privacy, even upon request. While the other three sites were willing to make accommodations if needed, these rooms were multi‐purpose and did not prioritise breastfeeding. None of the sites provided public information on requesting such accommodations. While the flooring, lighting and aesthetics were generally acceptable at all sites, none of them had suitable chairs for breastfeeding, and readily available drinking water was not provided. Only two sites had specific infant changing facilities.

In terms of breastfeeding policy, it should be acknowledged that none of the healthcare sites had BFHI status or had fully implemented the WHO/UNICEF BFHI Ten Steps for breastfeeding support. Findings revealed that the Primary Care Centre (PCC) was the only site acknowledging compliance with The Code. Neither of the two GP practices had a breastfeeding policy, nor the hospital and PCC had policies. The hospital acknowledged their practices were not aligned with evidence‐based standards. There were no policies or guidelines for new staff orientation, no visible breastfeeding policies and no records of staff education or training. However, all sites reported staff awareness of breastfeeding importance, and the hospital and PCC had training records within 6 months of healthcare professionals commencing employment, covering the BFHI Ten Steps. Breastfeeding information and promotion materials were available in the hospital and PCC. Inconsistent practices were noted, an obvious one being incomplete early skin‐to‐skin contact and rooming‐in at the hospital as that was not applicable in general practice or public health nursing settings.

### 3.2 Respondent Demographics and Questionnaire Results

Eighty‐five staff completed the questionnaire with 60% of respondents working in the hospital, 22.3% in a GP practice and 17.6% aligned to a Primary Care Centre (Table 1). More demographic information about age distribution, gender, role of participants and levels of experience are provided in Table 1. Findings demonstrated a significant association between healthcare setting and staff age categories, with a notable tendency for younger respondents to work in acute settings and those in the 30–39 age range in community settings (*χ*² (4) = 12.765, *p* < 0.05) (Table 2).

**Table 2:** Chi‐squared summary statistics.

Variable^1^	Acute setting	Community setting	*X* ^2^ value	df	*p* value
Age					
< 30	16 (31.4)	1 (2.9)	12.765	4	0.012* (two‐sided *p*)
30–39	9 (17.6)	12 (35.3)			
40–49	19 (37.3)	12 (35.3)			
50–59	6 (11.8)	7 (20.6)			
60+	1 (2.0)	2 (5.9)			
Total	51 (100)	34 (100)			
Gender					
Male	9 (18)	1 (3.1)	4.032	1	0.045* (two‐sided *p*)
Female	41 (82)	31 (96.9)			
Total	50 (100)	32 (100)			
Breast feeding training in the last 5 years					
Yes	25 (49)	9 (26.5)	4.322	1	0.038* (two‐sided *p*)
No	26 (51)	25 (73.5)			
Total	51 (100)	34 (100)			

*p < .05.

Gender distribution was also significant, with females predominating in both settings (Table 2) and a smaller male presence in the community (*p* < 0.05) (Table 2). Linear‐by‐linear associations confirmed these trends. No significant difference was observed in years served in current role between acute and community healthcare respondents (Table 3).

**Table 3:** *T*‐test summary statistics.

Variable	Setting	*n*	Mean (SD)	*t*	df	*p* value
Experience	Acute	51	9.31 (8.16)	−0.78	82	*p* = 0.938 (two‐sided *p*)
	Community	33	9.45 (7.96)			
VOCALISE TOOL						
VOCALISE Total Score	Acute	45	54.53 (9.39)	0.514	70	*p* = 0.609 (two‐sided *p*)
	Community	27	53.41 (8.31)			
1. When it comes to change, information is not circulated effectively on my ward.	Acute	47	3.94 (1.41)	0.644	78	*p* = 0.522 (two‐sided *p*)
	Community	33	3.73 (1.46)			
2. I feel confident when delivering new changes.	Acute	47	2.40 (1.01)	−0.331	78	*p* = 0.741 (two‐sided *p*)
	Community	33	2.48 (1.14)			
3. My whole team is regularly consulted about new ideas forward practices.	Acute	47	3.02 (1.33)	−0.524	78	*p* = 0.602 (two‐sided *p*)
	Community	33	3.18 (1.38)			
4. I'm too busy to keep up to date with information about the changes that are happening on my ward.	Acute	46	4.17 (1.16)	−0.701	77	*p* = 0.485 (two‐sided *p*)
	Community	33	4.36 (1.22)			
5. We can easily fit new changes in with our usual ward practices.	Acute	47	2.72 (1.12)	0.105	78	*p* = 0.917 (two‐sided *p*)
	Community	33	2.70 (1.10)			
6. I feel disheartened when others do not want to get involved in changes.	Acute	47	2.40 (1.21)	−0.351	77	*p* = 0.727 (two‐sided *p*)
	Community	32	2.50 (1.16)			
7. I think that managing risk is more important than delivering new changes.	Acute	47	3.13 (1.31)	−0.426	76	*p* = 0.671 (two‐sided *p*)
	Community	31	3.26 (1.34)			
8. Changes just increase my workload and make my life harder.	Acute	47	4.13 (1.21)	−0.117	76	*p* = 0.907 (two‐sided *p*)
	Community	31	4.16 (1.29)			
9. It is not clear how all changes that we are asked to make will really benefit my ward.	Acute	47	3.51 (1.32)	−1.555	76	*p* = 0.124 (two‐sided *p*)
	Community	31	3.97 (1.20)			
10. My teammates think that there is no point trying to implement some changes because they won't work.	Acute	47	3.62 (1.31)	−2.371	72.673	*p* = 0.20 (two‐sided *p*)
	Community	31	4.26 (1.06)			
11. I find it de‐motivating when new changes do not take patients' wishes into account.	Acute	46	2.39 (1.15)	−0.224	75	*p* = 0.823 (two‐sided *p*)
	Community	31	2.45 (1.18)			
12. I think that some staff would rather let others take the lead in making changes.	Acute	47	2.13 (1.15)	−1.323	76	*p* = 0.190 (two‐sided *p*)
	Community	31	2.48 (1.18)			
13. When some staff stop engaging with planned changes resistance spreads through my whole team.	Acute	47	2.85 (1.23)	−0.177	76	*p* = 0.860 (two‐sided *p*)
	Community	31	2.90 (1.33)			
14. I do not really understand how to deliver some of the changes that are suggested by the management.	Acute	47	3.83 (1.11)	−0.155	76	*p* = 0.877 (two‐sided *p*)
	Community	31	3.87 (1.20)			
15. Changes are audited to increase their consistent delivery on my ward.	Acute	47	2.81 (1.19)	−2.059	73	*p* = 0.43* (two‐sided *p*)
	Community	28	3.43 (1.37)			
16. I always challenge team members who are avoiding delivering new changes.	Acute	47	3.62 (1.34)	−1.159	74	*p* = 0.250 (two‐sided *p*)
	Community	29	3.97 (1.15)			
17. Inadequate staffing prevents changes being successful on my ward.	Acute	47	1.70 (1.02)	−3.297	76	*p* = 0.001** (two‐sided *p*)
	Community	31	2.55 (1.23)			
18. Poor leadership prevents changes happening on my ward.	Acute	47	3.21 (1.61)	−0.782	75	*p* = 0.437 (two‐sided *p*)
	Community	30	3.50 (1.50)			

*Note:* There was no statistically significant difference in mean total VOCALISE score between community and acute staff.

*p < 0.05, **p < 0.001.

### 3.3 Level of Engagement and Training

In terms of level of engagement supporting breastfeeding women and babies, 37.6% of participants were actively engaged and 46.5% had completed some form of breastfeeding training in the last 5 years. There was no significant difference in the level of engagement detected between acute and community staff. Non‐healthcare staff had not received any breastfeeding training, and staff at one GP surgery had also not undertaken any training in the previous 5 years. The types of breastfeeding training completed varied with most having completed the WHO Baby Friendly 6‐h training course (*n* = 14, 29.2%), nine participants had completed the 20‐h breastfeeding course and five were trained to International Board‐Certified Consultant (IBCLC) level. Respondents in acute care settings were significantly more likely to have completed breastfeeding training within the last 5 years (73.5%) compared to those in community settings (51%) (Table 2).

### 3.4 Staff Perceptions of Barriers to Change

The total VOCALISE scores ranged from 33 (positive) to 79 (negative). The total mean score of 54.11 (±8.96) fell within the positive range with 55% of respondents having a positive perspective of change implementation. Seventy‐two per cent (61/85) of respondents identified at least one perceived barrier to change with the top three barriers identified being staff shortages (*n* = 42, 68.9%), lack of communication (*n* = 24, 39.3%) and resistance to change (*n* = 19, 31.1%). Other commonly cited barriers related to availability of time and leadership.

There was no statistically significant difference in the mean total VOCALISE score between community and acute staff (Table 3). Subscale analysis of VOCALISE (Table 4) indicates that three quarters of all respondents as a group are highly motivated to engage in change but only one in three feel empowered and confident to do so.

**Table 4:** VOCALISE subscale analysis.

Total score	1–18	Positive range	Negative range	Ambivalent range
		18–54	72–108	55–71
		40 (55.6)	33 (1.4)	2 (43.0)
Powerlessness	4, 5, 7, 8, 9, 14, 17	Positive range	Negative range	Ambivalent range
		7–21	28–42	22–27
		23 (30.7)	13 (10.7)	39 (58.6)
Confidence	1, 2, 3, 10, 15, 16	Positive range	Negative range	Ambivalent range
		6–18	24–36	19–23
		25 (33.8)	12 (5.4)	38 (60.8)
De‐motivation	6, 11, 12, 13, 18	Positive range	Negative range	Ambivalent range
		5–15	20–30	16–19
		52 (73.3)	5(4.0)	15 (22.2)

*Note:* There were no significant differences between acute and community staff on these subscales.

A series of independent samples *t*‐tests was conducted to compare staff responses from acute and community healthcare settings on various aspects of change management as captured by VOCALISE (Table 3). This analysis found that overall, perceptions between the two main settings did not significantly differ on most items, with both acute and community staff expressing similar sentiments about the effectiveness of communication, confidence in change delivery and consultation on new ideas. However, there were a few notable exceptions. Item 10 revealed a statistically significant difference with community staff being less likely to agree that their teammates considered implementation of changes as futile (*p* = 0.020). Item 15, which dealt with the auditing of changes, showed that acute staff were less likely to disagree that changes would be subject to audit (*p* = 0.026, two sided). Finally, perceptions of staffing adequacy significantly differed; acute care staff were more likely to agree that inadequate staffing prevented successful changes (*p* < 0.001, two sided).

### 3.5 Staff Perceptions of Clinical Practice Guideline Implementation

Table 5 provides the descriptive statistics for the EBIQ dimensions of clinical experience, patient experience, research utilisation and context and facilitation. As per Bahtsevani and Idvall (2016), low scores on the 0–10 scale indicate low perceptions and those over the arbitrary marker of 5 indicate high perceptions. For all items relevant to clinical experience, patient experience, research utilisation and content of care and facilitation, the mean scores were consistently above the midpoint of five, demonstrating the perceived importance of all five dimensions to respondents in this study. Furthermore, 62.3% (53/85) of respondents indicated that they discussed and reflected on the importance of clinical experience in the workplace; 64.7% (55/85) on the importance of patient experience; 47.1% (40/85) on the importance of research utilisation and 48.2% (41/85) on the importance of context and facilitation.

**Table 5:** EBIQ descriptive statistics.

	0 (Low)	10 (High)	*n*	*M*	SD
Clinical experience	Clinical experience is discussed unsystematically without critical reflection	Clinical experience is discussed systematically with critical reflection	72	5.7	2.5
	Clinical experience of staff is not judged	Clinical experience of staff is judged	72	6.5	2.7
	Mutual understanding is lacking within my own health profession concerning the value of clinical experience	Mutual understanding exists within my own health profession concerning the value of clinical experience	74	6.1	2.7
	Clinical experience is not valued as a form of evidence	Clinical experience is valued as a form of evidence	74	6.5	2.8
	Clinical experience is valued as the only form of valid knowledge in decision‐making	Clinical experience is valued as one of several forms of valid knowledge in decision‐making	72	7.1	2.6
Patients experience	Patient biographies and experience are not used	Patient biographies and experience are used	73	6.7	2.5
	Patients are not involved in the planning of care actions	Patients are involved in the planning of care actions	72	6.5	2.9
	No partnership exists between patients and health professionals	A partnership exists between patients and health professionals	73	7.2	2.6
	Patients' experiences are not valued as a form of evidence	Patients' experiences are valued as a form of evidence	73	7.1	2.6
	Patients' experiences are valued as the only valid knowledge in decision‐making	Patients' experiences are valued as one of several forms of valid knowledge in decision‐making	72	6.9	2.6
Research utilisation	Current research is poorly conceived, designed, and/or executed	Current research is well conceived, designed, and executed	70	5.7	2.5
	Research is not valued as a form of evidence	Research is valued as a form of evidence	70	7.2	2.5
	Research is valued as the only form of valid knowledge in decision‐making	Research is valued as one of several forms of valid knowledge in decision‐making	70	7.1	2.4
	Research is viewed as certain and established knowledge	Research is viewed as knowledge whose importance must be appraised	71	7.7	1.9
Content and facilitation	The context is not receptive to change	The context is receptive to change	67	6.0	2.1
	The context is characterised by a culture that promotes a task‐driven organisation	The context is characterised by a culture that promotes a learning organisation	68	6.4	2.4
	The context is characterised by a culture that is unclear about values and beliefs	The context is characterised by a culture that is clear about prevailing values and beliefs	67	6.3	2.2
	The context is characterised by traditional (command and control) leadership	The context is characterised by transformational leadership	68	5.7	2.2
	The context is characterised by a leadership with autocratic and checking approach to learning, teaching, and managing	The context is characterised by a leadership with an enabling and empowering approach to learning, teaching, and managing	68	6.1	2.4
	Clinical, performance, economic, and experience evaluations rely on single rather than multiple methods	Multiple methods are used for clinical, performance, economic, and experience evaluations	69	6.2	2.3
	Absence of feedback concerning individual, team, and system performance	Presence of feedback on individual, team, and system performance	69	5.8	2.5
	The absence of facilitators or facilitation methods is inappropriate	Presence of facilitators, and appropriate facilitation methods	67	5.8	2.5
	The function and role of facilitator aims at doing for others (e.g., searching for research literature)	The function and role of facilitator aim at enabling others (e.g., teaching searching for literature)	67	6.1	2.1

Apart from one item regarding the perceived role of a facilitator, there was no significant difference in mean score on any item of the EBIQ for acute and community respondents. Community staff were significantly less likely to perceive the role of a facilitator as an enabler compared to acute staff as revealed by an independent *t*‐test (*p* = 0.05); however, the magnitude of the effect is modest with substantial overlap between the group's perceptions.

### 3.6 Staff Self‐Rated Competency

Sixty‐four per cent (54/85) of respondents answered at least one item on the awareness level section of the competency questionnaire; however, the frequency of responses to the items in the awareness section varied considerably. For example, 89.4% (42/47) of respondents could identify two ways to protect and support breastfeeding within the activities of their role; 63% (17/27) of respondents could name the policy relevant to breastfeeding in their workplace and 75.9% (41/54) considered breastfeeding beyond 6 months to have value. Technically those who completed this section of the competency questionnaire were not supposed to also complete the generalist section; however, 62.4% (53/85) of respondents did attempt at least part of the generalist section. The scores for each of the competency areas (knowledge, skills, behaviour and attitude) were reviewed. These scores were on a Likert scale from 1 to 5, where 1 = strongly agree and 5 = strongly disagree. Therefore, taking all scores below the midpoint (scores of 1, 2 and 2.5) for each domain was found to be more positive (or better) than those above the midpoint. On average, respondents scored below the midpoint on breastfeeding knowledge (median = 38, mid‐point is 47.5), skills (median = 41, mid‐point is 45), behaviours (median = 18.5 mid‐point is 22.5) and attitude (median = 4, mid‐point is 5).

The median rank attitude, knowledge, behaviour and skill scores were all higher in community‐based respondents than those from acute settings (Table 6). Due to smaller sample sizes and unequal variances for the between‐group comparisons, the non‐parametric Mann–Whitney *U* test was applied, and this revealed that the difference noted in self‐perceived attitude and knowledge was not statistically significant; however, in evaluating the perceived skills and behaviours between personnel in acute and community healthcare settings, a significant difference was identified for skills (*U* = 365.500, acute *n* = 32, community *n* = 17, *p* = 0.049) and behaviours (*U* = 360.0, acute *n* = 31, community *n* = 17, *p* = 0.037) (Table 6). The results suggest that individuals in the community setting displayed higher self‐perceived skill and behaviour rank scores compared to their counterparts in acute care.

**Table 6:** Competency assessment (adapted from Gallagher tool).

		*N*	Mean rank	Mann Whitney *U*	*p* value
Attitude	Acute	32	23.75	376.00	*p* = 0.141 (two sided)
	Community	19	29.79		
Knowledge	Acute	32	23.38	324.00	*p* = 0.274 (two sided)
	Community	17	28.06		
Skills	Acute	32	22.08	365.5	*p* = 0.049* (two sided)
	Community	17	30.50		
Behaviour	Acute	31	21.39	360.00	*p* = 0.037* (two sided)
	Community	17	30.18		

* The results suggest that individuals in the community setting displayed higher self‐perceived skill and behaviour rank scores.

## 4 Discussion

The primary goal of this baseline situational analysis was to augment the development of effective and sustainable exclusive breastfeeding strategies.

The VOCALISE score as a metric to gauge staff perceptions towards change was useful and the results indicated that the total mean score fell within the positive range, suggesting that a majority of the staff held a positive perception towards the implementation of change. This is indicative of a workforce that is, overall, open and receptive to new initiatives and practices related to breastfeeding support. However, a smaller proportion felt empowered and confident to engage in change. This gap between motivation and empowerment highlights the need for supportive mechanisms that equip staff with the necessary skills and confidence to implement change effectively, again linking to the facilitation element of PARIHS. Community staff, despite having lower attendance rates at training in the last 5 years, were found to have no difference in knowledge and attitudes, but it was found that their ratings for skills and behaviour were significantly greater. This may be because healthcare staff in the community in this study are older than their hospital counterparts. But besides age, there may be other factors on which data were not collected which could be playing a role. They may be potentially more experienced or have more time available to support women to enhance their skills.

The results further indicate the value of separating the elements of competency, that is, knowledge, attitude, skill and behaviour. This approach supports the current skills‐focused, outcome‐based National Infant Feeding Education Programme (NIFEP) in Ireland (HSE 2022). The NIFEP was informed by work commissioned by the Department of Health, culminating in the published evidence of the need for a skill‐based approach (Mulcahy et al. 2022).

This study identified a significant level of engagement among healthcare staff in supporting breastfeeding mothers and infants with distinct differences between acute and community settings. Healthcare staff in acute care demonstrated higher completion rates of recent breastfeeding training compared to community settings, highlighting the need for a comprehensive approach to training across all healthcare settings. This aligns with existing literature that found variability in breastfeeding support training across healthcare professions (Hookway and Brown 2023), impacting their ability to effectively promote and support exclusive breastfeeding. Key barriers identified included staff shortages, lack of communication and resistance to change, with these challenges consistently present across both settings with staff shortage concerns emphasised more among acute care respondents. Even though there were staff who were initially unwilling to participate and nearly a third were resistant among those who did take part, the study design and the way it was implemented (reported elsewhere in Lehane et al. 2024) illustrated how all staff were kept appraised of study developments and a communication book was kept in each ward or unit accessible to all. The PARIHS framework emphasises the importance of adapting EBPs to the specific contexts of each healthcare setting. In our study, consideration of context through the delineation of differences between settings was necessary to ascertain such a nuanced perspective. The physical environment assessment revealed inadequacies in both acute and community settings, notably the lack of designated breastfeeding areas and suboptimal facilities. This requires attention to infrastructural changes in line with the Baby‐Friendly Hospital Initiative standards (WHO and UNICEF 2018). Similar strategies are necessary to address staff differences and consequent learning and competency needs.

The study's strengths include its comprehensive approach and inclusivity of healthcare and non‐healthcare staff. Limitations, however, stem from its regional focus and the impact of the Covid pandemic on environmental factors, potentially affecting the generalisability of findings. As with studies using purposive sampling methods, this study also would have encountered some potential selection bias and that could impact the generalisability of the findings. While the study settings are typical of Irish maternity and post‐natal services, we would make no claims on the generalisability of the issue. However, we would recommend repeating similarly designed studies in other settings or counties. The inclusion of GP medical centres while novel, added unique complexities particularly regarding the application of existing breastfeeding audit tools. Future research should explore a broader range of settings and include longitudinal studies.

## 5 Conclusion

This multi‐component baseline situational analysis provides a solid foundation to advance environmental, cultural, practical and policy changes required to successfully promote exclusive breastfeeding within acute and community care. The primary aim of this baseline analysis is to increase the effectiveness and sustainability of exclusive breastfeeding strategies. This would in turn lead to better infant and maternal health outcomes. One of the tools used, the VOCALISE score, was a metric to quantify staff perceptions towards change. This was very useful as it provided an idea of how majority of the staff held a positive perception towards the implementation of change. This meant that overall, the workforce is open to new initiatives. Also, it is observed that only a small number of personnel who felt confident about change would indicate that support mechanisms could be put in place so that the change can be brought about effectively. The forthcoming stages of this research involve the development of a multi‐level, multi‐disciplinary plan framed by a Participatory Action Research approach, followed by the evaluation of changes in perceived skill, knowledge, behaviour and attitude/practice, policy and culture.

## Author Contributions

All authors contributed to the journal article. P.L.‐W. and H.M. were co‐principal investigators. P.L.‐W., H.M., M.O'.D., E.L., C.B., R.O'.C. and M.M. designed the research study. H.M. and M.O'.D. worked on several of the tools. L.C. administered, collated and inputted data from the questionnaires supported by M.O'.D. M.O'.D. carried out preliminary analysis, E.M.C. collated all data and carried out further analysis and K.M.L. carried out final analysis and compiled journal paper for publication.

## Conflicts of Interest

The authors declare no conflicts of interest.

## Data Availability

The authors have nothing to report.

## References

[mcn13778-bib-0001] Bahtsevani, C. , and E. Idvall . 2016. “To Assess Prerequisites Before an Implementation Strategy in an Orthopaedic Department in Sweden.” Orthopaedic Nursing 35: 100–107. https://www.ingentaconnect.com/content/wk/nor/2016/00000035/00000002/art00009. 27028686 10.1097/NOR.0000000000000225

[mcn13778-bib-0002] Bahtsevani, C. , A. Willman , A. Khalaf , and M. Östman . 2008. “Developing an Instrument for Evaluating Implementation of Clinical Practice Guidelines: A Test‐Retest Study.” Journal of Evaluation in Clinical Practice 14: 839–846. 18331325 10.1111/j.1365-2753.2007.00916.x

[mcn13778-bib-0003] Baker, P. , J. P. Smith , A. Garde , et al. 2023. “The Political Economy of Infant and Young Child Feeding: Confronting Corporate Power, Overcoming Structural Barriers, and Accelerating Progress.” Lancet 401: 503–524. 10.1016/s0140-6736(22)01933-x. 36764315

[mcn13778-bib-0004] Baum, F. , C. MacDougall , and D. Smith . 2006. “Participatory Action Research.” Journal of Epidemiology & Community Health 60: 854–857. 16973531 10.1136/jech.2004.028662 PMC2566051

[mcn13778-bib-0005] Department of Health . 2016. “Creating a Better Future Together: National Maternity Strategy 2016–2026.” www.gov.ie.

[mcn13778-bib-0006] Department of Health . 2019. *Sláintecare Action Plan 2019*. Dublin, Ireland: Author. https://www.gov.ie/en/publication/109e2b-slaintecare-action-plan-2019/.

[mcn13778-bib-0007] Department of Health . 2021. *Sláintecare Implementation Strategy and Action Plan 2021–2023*. Dublin, Ireland: Author. https://www.gov.ie/en/publication/6996b-slaintecare-implementation-strategy-and-action-plan-2021-2023/.

[mcn13778-bib-0008] Department of Health . 2023. “Breastfeeding in a Healthy Ireland—Health Service Executive Action Plan 2016–2021.” Implementation Progress Report.

[mcn13778-bib-0009] Department of Health and Children . 2005. “Breastfeeding in Ireland: A Five‐Year Strategic Action Plan.” https://www.lenus.ie/handle/10147/267732.

[mcn13778-bib-0010] Duan, Y. , A. Iaconi , J. Wang , et al. 2022. “Conceptual and Relational Advances of the PARIHS and i‐PARIHS Frameworks Over the Last Decade: A Critical Interpretive Synthesis.” Implementation Science 17: 78. 10.1186/s13012-022-01254-z. 36476376 PMC9730581

[mcn13778-bib-0011] Gallagher, L. , K. Muldoon , and D. McGuinness . 2015. Competence Framework for Breastfeeding Support. The University of Dublin, Trinity College Dublin. https://www.hse.ie/file-library/competence-framework-for-breastfeeding-support.pdf.

[mcn13778-bib-0801] Gavine, A. , A. McFadden , S. MacGillivray , and M. J. Renfrew . 2017. “Evidence Reviews for the Ten Steps to Successful Breastfeeding Initiative.” Journal of Health Visiting 5, no. 8: 378–380.

[mcn13778-bib-0013] Graham, I. , J. Tetroe , and A. Pearson . 2014. Turning Knowledge Into Action: Practical Guidance on How to Do Integrated Knowledge Translation Research. Philadelphia: Book 21.

[mcn13778-bib-0014] Harrison, M. B. , and I. D. Graham . 2012. “Roadmap for a Participatory Research‐Practice Partnership to Implement Evidence.” Worldviews on Evidence‐Based Nursing 9: 210–220. 22672620 10.1111/j.1741-6787.2012.00256.x PMC3791554

[mcn13778-bib-0016] Hookway, L. , and A. Brown . 2023. “The Lactation Skill Gaps of Multidisciplinary Paediatric Healthcare Professionals in the United Kingdom.” Journal of Human Nutrition and Dietetics 36: 848–863. 10.1111/jhn.13172. 36992632

[mcn13778-bib-0017] HSE . 2016. “Breastfeeding in a Healthy Ireland Health Service Breastfeeding Action Plan 2016–2021.” https://www.breastfeeding.ie/Uploads/breastfeeding-in-a-healthy-ireland.pdf.

[mcn13778-bib-0018] HSE . 2022. “National Standards for Infant Feeding in Maternity Services.” Dublin: Ireland. https://www.hse.ie/eng/about/who/acute-hospitals-division/woman-infants/infant-feeding/.

[mcn13778-bib-0020] Kitson, A. , G. Harvey , and B. McCormack . 1998. “Enabling the Implementation of Evidence‐Based Practice: A Conceptual Framework.” Quality and Safety in Health Care 7: 149–158. 10.1136/qshc.7.3.149 PMC2483604 10185141

[mcn13778-bib-0021] Kitson, A. L. , and G. Harvey . 2016. “Methods to Succeed in Effective Knowledge Translation in Clinical Practice.” Journal of Nursing Scholarship 48: 294–302. 10.1111/jnu.12206. 27074390

[mcn13778-bib-0022] Kitson, A. L. , J. Rycroft‐Malone , G. Harvey , B. McCormack , K. Seers , and A. Titchen . 2008. “Evaluating the Successful Implementation of Evidence into Practice Using the Parihs Framework: Theoretical and Practical Challenges.” Implementation Science 3: 1. 10.1186/1748-5908-3-1. 18179688 PMC2235887

[mcn13778-bib-0023] Laker, C. , F. Callard , C. Flach , P. Williams , J. Sayer , and T. Wykes . 2014. “The Challenge of Change in Acute Mental Health Services: Measuring Staff Perceptions of Barriers to Change and Their Relationship to Job Status and Satisfaction Using a New Measure (VOCALISE).” Implementation Science 9: 23. 10.1186/1748-5908-9-23. 24555496 PMC4016533

[mcn13778-bib-0024] Lehane, E. , C. Buckley , H. Mulcahy , et al. 2024. “Evaluating the Process of Practice Enhancement for Exclusive Breastfeeding (PEEB): A Participatory Action Research Approach for Clinical Innovation.” International Breastfeeding Journal 19, no. 1: 39. 38822371 10.1186/s13006-024-00648-7 PMC11140990

[mcn13778-bib-0025] Lockwood, C. , M. Stephenson , L. Lizarondo , J. van Den Hoek , and M. Harrison . 2016. “Evidence Implementation: Development of an Online Methodology From the Knowledge‐to‐Action Model of Knowledge Translation.” International Journal of Nursing Practice 22: 322–329. 10.1111/ijn.12469. 27562662

[mcn13778-bib-0026] Meloncelli, N. , A. Barnett , and S. de Jersey . 2020. “An Implementation Science Approach for Developing and Implementing a Dietitian‐Led Model of Care for Gestational Diabetes: A Pre‐Post Study.” BMC Pregnancy and Childbirth 20: 661. 10.1186/s12884-020-03097-4. 33143693 PMC7607700

[mcn13778-bib-0027] Mulcahy, H. , L. F. Philpott , M. O'Driscoll , R. Bradley , and P. Leahy‐Warren . 2022. “Breastfeeding Skills Training for Health Care Professionals: A Systematic Review.” Heliyon 8: e11747. 10.1016/j.heliyon.2022.e11747. 36468118 PMC9708688

[mcn13778-bib-0028] National Women and Infants Health Programme (NWIHP) . 2021. “Irish Maternity Indicator System Nation Report 2020.” https://www.hse.ie/eng/about/who/acute-hospitals-division/woman-infants/national-reports-on-womens-health/irish-maternity-indicator-system-national-report-2020.pdf.

[mcn13778-bib-0029] Niemeyer Hultstrand, J. , E. Engström , M. Målqvist , T. Tydén , N. Maseko , and M. Jonsson . 2020. “Evaluating the Implementation of the Reproductive Life Plan in Disadvantaged Communities: A Mixed‐Methods Study Using the i‐PARIHS Framework.” PLoS One 15, no. 9: e0236712. 10.1371/journal.pone.0236712. 32915798 PMC7485818

[mcn13778-bib-0030] Parmelli, E. , G. Flodgren , F. Beyer , N. Baillie , M. E. Schaafsma , and M. P. Eccles . 2011. “The Effectiveness of Strategies to Change Organisational Culture to Improve Healthcare Performance: A Systematic Review.” Implementation Science 6: 33. 10.1186/1748-5908-6-33. 21457579 PMC3080823

[mcn13778-bib-0031] Pérez‐Escamilla, R. , C. Tomori , S. Hernández‐Cordero , et al. 2023. “Breastfeeding: Crucially Important, but Increasingly Challenged in a Market‐Driven World.” Lancet 401: 472–485. 10.1016/s0140-6736(22)01932-8. 36764313

[mcn13778-bib-0033] Rollins, N. , E. Piwoz , P. Baker , et al. 2023. “Marketing of Commercial Milk Formula: A System to Capture Parents, Communities, Science, and Policy.” Lancet 401: 486–502. 10.1016/s0140-6736(22)01931-6. 36764314

[mcn13778-bib-0034] Rycroft‐Malone, J. , A. Kitson , A. Harvey , et al. 2002. “Ingredients for Change: Revisiting a Conceptual Framework.” Quality and Safety in Health Care 11: 174–180. 12448812 10.1136/qhc.11.2.174 PMC1743587

[mcn13778-bib-0035] Rycroft‐Malone, J. 2004. “The PARIHS Framework—A Framework for Guiding the Implementation of Evidence‐Based Practice.” Journal of Nursing Care Quality 19, no. 4: 297–304. 15535533 10.1097/00001786-200410000-00002

[mcn13778-bib-0036] Rycroft‐Malone, J. , G. Harvey , K. Seers , A. Kitson , B. McCormack , and A. Titchen . 2004. “An Exploration of the Factors That Influence the Implementation of Evidence into Practice.” Journal of Clinical Nursing 13: 913–924. 15533097 10.1111/j.1365-2702.2004.01007.x

[mcn13778-bib-0037] Strid, E. N. , L. Wallin , and Y. Nilsagård . 2022. “Implementation of a Health Promotion Practice Using Individually Targeted Lifestyle Interventions in Primary Health Care: Protocol for the “Act in Time” Mixed Methods Process Evaluation Study.” JMIR Research Protocols 11, no. 8: e37634. 10.2196/37634. 35984700 PMC9440414

[mcn13778-bib-0039] WHO and UNICEF . 2018. Protecting, Promoting and Supporting Breastfeeding in Facilities Providing Maternity and Newborn Services: Implementing the Revised Baby‐Friendly Hospital Initiative 2018. Geneva: World Health Organization and the United Nations Children's Fund (UNICEF). https://iris.who.int/bitstream/handle/10665/272943/9789241513807-eng.pdf?sequence=19.

[mcn13778-bib-0042] World Health Organization . 1981. “International Code of Marketing of Breastmilk Substitutes and Relevant WHA Resolutions.” https://www.who.int/publications/i/item/9241541601.

[mcn13778-bib-0043] World Health Organization . 2001. “The Optimal Duration of Exclusive Breastfeeding: A Systematic Review.” WHO/NHD/01.08; WHO/FCH/CAH/01.23. https://www.who.int/publications-detail-redirect/WHO-NHD-01.08.

[mcn13778-bib-0044] World Health Organization . 2009. “Baby‐Friendly Hospital Initiative: Revised, Updated and Expanded for Integrated Care. Section 1 to 4.” https://www.who.int/publications/i/item/9789241594950. 23926623

